# Stillbirths at Term: Case Control Study of Risk Factors, Growth Status and Placental Histology

**DOI:** 10.1371/journal.pone.0166514

**Published:** 2016-12-09

**Authors:** Federico Mecacci, Caterina Serena, Laura Avagliano, Mauro Cozzolino, Eleonora Baroni, Marianna Pina Rambaldi, Serena Simeone, Francesca Castiglione, Gian Luigi Taddei, Gaetano Bulfamante

**Affiliations:** 1 Department of Sciences for the Health of Women and Children, Careggi Hospital, Florence, Italy; 2 Department of Health Sciences, San Paolo Hospital Medical School University of Milan, Milan, Italy; 3 Department of Biomedical, Experimental and Clinical Sciences-Division of Obstetrics and Gynaecology, University of Florence, Florence, Italy; 4 Department of Biomedicine, Careggi Hospital, Florence, Italy; Universitat de Barcelona, SPAIN

## Abstract

**Objective:**

To investigate the proportion of stillbirths at term associated with abnormal growth using customized birth weight percentiles and to compare histological placental findings both in underweight stillborn fetuses and in live births.

**Methods:**

A retrospective case-control study of 150 singleton term stillbirths. The livebirth control groups included 586 cases of low-risk pregnancies and 153 late fetal growth restriction fetuses. Stillbirths and livebirths from low-risk pregnancies were classified using customized standards for fetal weight at birth, as adequate for gestational age (AGA; 10-90^th^ percentile), small (SGA; <10^th^ percentile) or large for gestational age (LGA; >90^th^ percentile). Placental characteristics in stillbirth were compared with those from livebirths using four categories: inflammation, disruptive, obstructive and adaptive lesions.

**Results:**

There was a higher rate of SGA (26% vs 6%, p<0.001) and LGA fetuses (10.6% vs 5.6%, p<0.05) in the stillbirth group. Among stillbirth fetuses, almost half of the SGA were very low birthweight (≤3°percentile) (12% vs 0.3%, p<0.001). The disruptive (7.3% vs 0.17%;p<0.001), obstructive (54.6% vs 7.5%;p<0.001) and adaptive (46.6% vs 35.8%;p<0.001) findings were significantly more common in than in livebirth-low risk. Placental characteristics of AGA and SGA stillbirth were compared with those of AGA and FGR livebirth. In stillbirths-SGA we found a higher number of disruptive (12.8% vs 0%; p<0.001), obstructive (58.9% vs 23.5%;p<0.001) and adaptive lesions (56.4% vs 49%; p 0.47) than in livebirth-FGR.

**Conclusion:**

The assessment of fetal weight with customized curves can identify fetuses which have not reached their genetically determined growth potential and are therefore at risk for adverse outcomes. Placental evaluation in stillbirths can reveal chronic histological signs that might be useful to clinical assessment, especially in underweight fetuses.

## Introduction

Intrauterine fetal death is a most tragic complication of pregnancy, and occurs at an estimated rate of 3.1/1000 deliveries in developed countries and 30/1000 in developing countries. Over 2.6 million stillbirths after 28 weeks of gestation or including fetuses weighting more than 1000g occur each year worldwide and 80% of these occur at the end of pregnancy [1].

Case reviews indicate that many late fetal losses are associated with a failure in identifying risk factors. Underestimating these hazard leads to a lack of appropriate standard of care for pregnancies at risk for stillbirth [2]. Abnormal fetal growth is one of the main risk factors for stillbirth [3]: both small for gestational age (SGA) and large for gestational age (LGA) fetuses have been associated with increased risk of intrauterine death. Among fetuses destined to be stillborn, customized norms, rather than population standards, seem to perform best both in detecting a higher rate of SGA and LGA as well as in identifying SGA fetuses at risk of death [4]. Previous studies have shown that stillbirths are frequently associated with unrecognized growth abnormality [3,5]: if the fetuses fail to reach their growth potential, risk of stillbirth is increased 5 to 10 fold [6].

The placenta is a fundamental substrate for the development and evolution of pregnancy. Placental abnormalities affect several cases of intrauterine fetal death [7] and histological examination is strongly recommended by several international guidelines [8–11] also to interpret unfavorable pregnancy outcomes even in cases apparently occurring without clinical cause. However, despite placental examination appearing to be important in cases of intrauterine fetal death, a recent systematic review failed to identify a specific role of the various placental lesions in stillbirth [12].

We aimed to investigate the proportion of stillbirths at term associated with abnormal growth using customized percentiles for birthweight, and to compare histology of the placentas of underweight stillborn fetuses and two control populations, live born from low-risk pregnancy and live born with late fetal growth restriction (FGR).

## Material and Methods

This was a retrospective case-control study on all singleton stillbirths delivered between January 2009 and May 2015 in two Tertiary University Hospitals in the North of Italy (Careggi Hospital in Florence and San Paolo Hospital in Milan). The study was exempt from Institutional Review Board approval because placental histology and obstetric and neonatal outcomes were collected as part of clinical management. Patients signed informed consent for the clinical investigations and use of the results for scientific analysis according to privacy laws and human rights.

### Patient selection and data collection

Inclusion criteria for cases were singleton antepartum fetal death at or after 37 weeks of gestation. Exclusion criteria were major congenital anomalies and/or abnormal karyotype and maceration at post-mortem evaluation. We defined two control groups. The first included women who attended the low-risk antenatal clinics during the study period, had no relevant obstetric or medical history, and had an uncomplicated pregnancy with a live birth at term. The second control group included pregnancies with late fetal growth restriction (FGR) diagnosed sonographically after 34 weeks during the last year, and monitored until delivery in the Regional Reference Center for high-risk pregnancy, Careggi Hospital, Florence. FGR was defined as any of the following: abdominal circumference <10^th^ percentile, sonographically estimated fetal weight <10^th^ percentile according to Hadlock’s ultrasound norms, or abdominal circumference growth rate <11 mm in a 14-day interval [13]. FGR fetuses were delivered either spontaneously or iatrogenically by induction of labour or caesarean section according to the latest guidelines [14–15].

For each case, clinical data were recorded, including maternal ethnicity, age, parity, level of education, pre-pregnancy body mass index, smoking habits, gestational age at delivery, and birthweight and gender of the neonate.

Each case of stillbirth underwent a thorough evaluation according to a comprehensive protocol as previously published [16,17]. Briefly, the protocol included evaluation of maternal blood pressure, maternal blood testing for inherited and acquired thrombophilia, thyroid function, indirect Coombs test, screening for diabetes, serology for TORCH, placental pathology examination, fetal autopsy, fetal chromosomal analysis (from amniotic fluid or blood or fascia lata sampling). Maternal genital cultures and neonatal swabs were also performed for microbiological analysis.

### Evaluation criteria for neonatal fetal weight

Stillbirths and controls were classified on the basis of birthweight using customized percentiles, which included adjustment for maternal pre-pregnancy weight, height, parity and ethnicity as previously reported [5], using the Gestation Related Optimal Weight software (GROW version 6.7.5, 2014, www.gestation.net). The fetal age was the date of delivery for liveborn and stillborn, using menstrual dating criteria or ultrasound criteria when the last menstrual period was unknown, or in case of non-agreement with ultrasound dating (according to the Italian Society of Ultrasound in Obstetrics Guidelines [18]). For stillbirths, excluding macerated fetuses at birth, we considered the birthweight at in the delivery room and not that at the autopsy. Birthweight was considered appropriate for gestational age (AGA) between the 10^th^ and the 90^th^ percentile; the fetuses were otherwise categorized as small for gestational age, SGA (birthweight below 10^th^ percentile) or as large for gestational age, LGA (birthweight above 90^th^ percentile).

### Placental samples collection and histological classification

Placental macroscopic inspection of the maternal and fetal plates, umbilical cord and membranes was carried out as previously reported [19]. Placental sampling was conducted according to international criteria [20]. Briefly, after trimming the umbilical cord and membrane, the placental disc was cut in 1cm thick slices. At least one free membrane roll, one section of the umbilical cord and three conventional full-thickness sections of grossly unremarkable placental parenchyma were sampled for histological examination. All samples were collected by expert fetal and neonatal pathologists using standardized instructions to ensure quality in enrolment sampling and specimen processing. Formalin-fixed paraffin embedded placental tissue samples were processed for histopathological analysis and standard 4 μm thick tissue sections were stained with hematoxylin and eosin and examined at light microscopy by expert pathologists blinded for fetal growth characteristics. Placental lesions were identified through microscopy study according to standard international references [21,22]. Histological placental findings were classified as previously reported using four categories [23,24]: lesions of inflammation, disruptive lesions, obstructive lesions and adaptive lesions (Table 1).

**Table 1:** Patterns of histologic findings.

**Inflammation lesions**	Severe Acute Chorioamnionitis
	Funisitis
	Villitis of unknown etiology
**Disruptive lesions**	Abruptio placentae
	Rupture of vasa praevia
	Feto-maternal haemorrhage
**Obstructive lesions**	Infarction (>20% of the parenchyma)
	Massive fibrin deposition
	Diffuse villous edema
	Decidual vasculopathy
	Fetal thrombotic vasculopathy
**Adaptive lesions**	Incremented syncytial knots
	Chorangiosis
	Villous branching anomalies

### Statistical analysis

For categorical variables, frequencies and percentages were reported, means and standard deviations (±SD) for continuous variables. Differences between groups were analyzed with Student’s t test, Fisher exact test or chi-square test when appropriate. An a priori two-tailed level of significance was set at the 0.05 level.

## Results

There were 165 term stillbirths during the period of the study, of which 15 fetuses were excluded because of maceration, leaving 150 to be included in the study group. Two control groups were included:

- 586 livebirths from 600 low-risk uneventful singleton term pregnancies monitored at two low-risk clinics during the same period (14 cases were excluded due to incomplete data).
- 153 livebirth cases of late FGR diagnosed sonographically after 34 weeks during the last year.

Patient characteristics are summarized in Table 2.

**Table 2:** Patient characteristics.

Main characteristics	Stillbirth (n. 150)	Livebirth low risk (n. 586)	Livebirths FGR (n.153)	p1 value	p2 value	p3 value
**Maternal age (years) mean±SD**	31.8 ±5.0	30.9 ±5.02	33.62± 5.06	n.s.	n.s	n.s
**BMI (Kg/m**^**2**^**) mean±SD**	24.4 ±5.2	22.4 ±3.98	25.2±5.18	<0.001	n.s.	<0.001
**Obesity (BMI ≥30)**	(22) 14.6%	(30) 5.1%	(3) 1.9%	<0.001	<0.001	n.s
**Nulliparity**	(89) 59.3%	(252) 43%	(99) 64.7%	<0.001	n.s.	<0.001
**Education <8 ys**	(28) 18.6%	(42) 7.2%	(11) 7.1%	<0.001	<0.05	n.s.
**Smoking status-Smokers**	(16) 10.6%	(9) 1.5%	(17) 11.1%	<0.001	n.s	<0.001
**European ethnicity**	(120) 80%	(464) 79.2%	(142) 92.81%	n.s	<0.001	<0.001
**Chronic hypertension**	(0) 0%	(0) 0%	(3) 1.9%	n.s	n.s.	<0.001
**Diabetes mellitus I/II**	(5) 3.3%	(0) 0%	(0) 0%	<0.001	<0.05	n.s
**TORCH positivity**	(1) 0.6%	(0) 0%	(0) 0%	<0.05	n.s.	n.s.
**Inherited/acquired thrombophilia**	(13) 8.6%	(0) 0%	(14) 9.1%	<0.001	n.s.	<0.001
**Thyroid disease**	(4) 2.6%	(0) 0%	(16) 10.4%	<0.001	<0.05	<0.001
**Preeclampsia**	(3) 2%	(0) 0%	(0) 0%	<0.05	n.s.	n.s.

SD: standard deviation BMI: body mass index. P1: stillbirth vs livebirth from low-risk pregnancy, P2: stillbirth vs livebirth with late FGR, P3: livebirth from low-risk pregnancy vs livebirth with late FGR

Mean maternal age at conception was similar in the three groups (31.8 ±5.0 vs 30.9 ±5.02 vs 33.62± 5.06 years, respectively). There was a higher incidence of obesity (body mass index, BMI ≥30) in the stillbirth group than in both livebirth-low risk and livebirth-FGR groups (14.6% vs 5.1% vs 1.9). In the stillbirth group and livebirth-FGR group, women were more frequently smokers than in livebirth-low risk (10.6% and 11.1% vs 1.5%). Neonatal characteristics of all pregnancies are summarized in Table 3.

**Table 3:** Neonatal characteristics.

Main characteristics	Stillbirth (n. 150)	Livebirth-low risk (n. 586)	Livebirth FGR (n.153)	p1 value	p2 value	p3 value
**Gestational age at delivery (weeks)**	38.5 ±1.3	39.4±1.2	39.3±1.72	*n*.*s*	*n*.*s*	*n*.*s*
**GA ≥40 weeks**	(14) 9.3%	(235) 40.1%	(47) 30.7%	<0.001	<0.001	<0.05
**Neonatal birthweight (grams)**	3047 ±577.8	3330 ±314.3	2507.27±329.15	<0.001	<0.001	<0.001
**AGA according to customized centiles**	(95) 63.4%	(518) 88.4%	-	<0.001	-	-
**SGA according to customized centiles**	(39) 26%	(35) 6.0%	(153) 100%	<0.001	<0.001	<0.001
**Severe SGA (Birthweight ≤3° percentile)**	(18)12%	(2) 0.3%	(84) 54.2%	<0.001	<0.001	<0.001
**LGA according to customized centiles**	(16) 10.6%	(33) 5.6%	-	< 0.05	-	-
**Neonatal gender: Male**	(77) 51.3%	(304) 51.9%	(68) 44.4%	*n*.*s*	*n*.*s*	*n*.*s*
**Neonatal gender: Female**	(73) 48.7%	(282) 48.1%	(85) 55.6%	*n*.*s*	*n*.*s*	*n*.*s*

P1: stillbirth vs livebirth from low-risk pregnancy; P2: stillbirth vs livebirth with late FGR; P3: livebirth from low-risk pregnancy vs livebirth with late FGR

**Fig 1:**
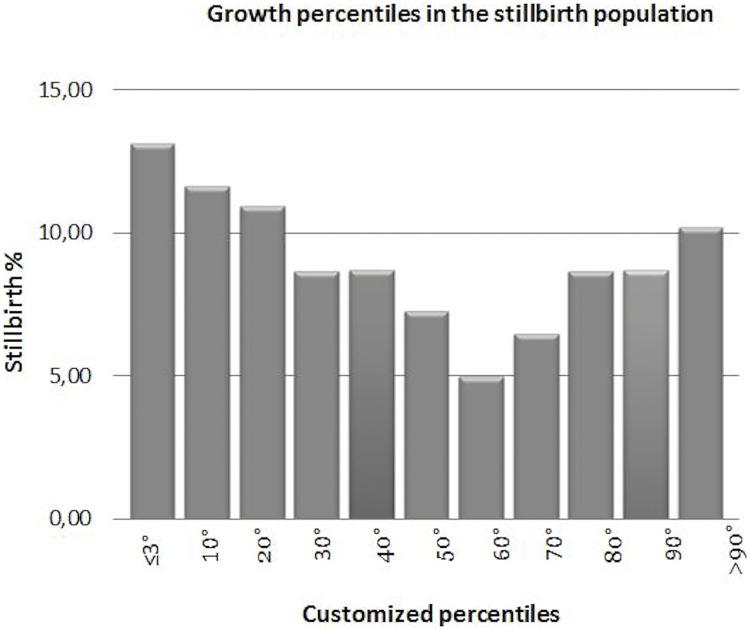
Birthweight percentile by customized percentiles in stillbirth population.

Gestational age did not significantly differ in all groups (38.5±1.3 vs 39.4±1.2 vs 39.3±1.72). Birthweight was lower in the livebirth-FGR than the stillbirth and livebirth-low risk groups (2507.27±329.15 vs 3330 ±314.3 in livebirth-low risk vs 3047 ±577.8 in stillbirth; p<0.001). The rate of neonatal gender was not significantly different in the three groups. In the stillbirth group, fetal growth was classified as abnormal (<10^th^ or >90^th^ percentiles) in 55 (36.6%) and normal in 95 (63.4%) of the cases. Distribution of growth percentiles in the stillbirth population is shown in Fig 1.

Customized percentiles show a U-shaped distribution with increased proportions of SGA, especially ≤3°percentile, and LGA fetuses. Customized percentiles identified a higher rate of SGA and LGA in the stillbirths compared to the livebirth-low risk group, with 26% of stillbirths vs 6% of livebirth being SGA (p<0.001). Almost half of these had a very low birthweight (≤3°percentile) (12% vs 0.3%, p<0.001). Moreover, in the stillbirth group we found a double rate of LGA fetuses (10.6% vs 5.6%, p<0.05). SGA pregnancies were associated with a 5.5-fold increased risk of stillbirth compared to AGA using customized standards and severe SGA (birthweight ≤3°percentile) was associated with a 39-fold increased risk of death. LGA fetuses were associated with a 1.99-fold increased risk of stillbirth at term using customized curves.

Placental characteristics are summarized in Table 4.

**Table 4:** Placental characteristics in cases and controls.

Placental lesions	Stillbirth (n. 150)	Livebirth-Low risk (n.586)	Livebirth-FGR (n.153)	p1 value	p2 value	p3 value
**Inflammatory**	(11) 7.3%	(29) 4.9%	(16) 10.4%	n.s.	n.s.	<0.05
**Disruptive**	(11) 7.3%	(1) 0.17%	(0) 0%	<0.001	<0.05	n.s.
**Obstructive**	(82) 54.6%	(44) 7.5%	(36) 23.5%	<0.001	<0.001	<0.001
**Adaptive**	(70) 46.6%	(210) 35.8%	(75) 49%	<0.05	n.s.	<0.05

p1: stillbirth vs livebirth from low-risk pregnancy; p2: stillbirth vs livebirth with late FGR; p3: livebirth from low-risk pregnancy vs livebirth with late FGR.

Histological findings are not mutually exclusive, more than one lesion may coexist in each case.

Placental lesions occurred more frequently in the stillbirth than the livebirth-low risk group: disruptive (7.3% vs 0.17%;p<0.001), obstructive (54.6% vs 7.5%;p<0.001) and also adaptive (46.6% vs 35.8%;p<0.001) findings were significantly more common for stillbirths, regardless of birthweight percentile.

To investigate the relationship between abnormal fetal weight and stillbirth, we subdivided the stillbirth population in AGA or SGA subgroups and we compared their placental characteristics with those of livebirth-AGA and livebirth-FGR (Table 5).

**Table 5:** Placental characteristics in AGA and SGA stillbirth vs AGA and SGA livebirth.

Placental lesions	Stillbirth-AGA (n. 95)	Stillbirth- SGA (n.39)	Livebirth-AGA(n.518)	Livebirth- FGR (n.153)	p1 value	p2 value	p3 value
**Inflammatory**	(10) 10.5%	(3) 7.7%	(24) 4.6%	(16) 10.4%	<0.05	n.s.	<0.05
**Disruptive**	(5) 5.2%	(5) 12.8%	(0) 0%	(0) 0%	<0.001	<0.05	n.s.
**Obstructive**	(44) 46.3%	(23) 58.9%	(19) 3.6%	(36) 23.5%	<0.001	<0.001	<0.001
**Adaptive**	(9) 9.4%	(22) 56.4%	(181) 34.9%	(75) 49%	<0.001	<0.001	<0.001

p1: stillbirth-AGA vs livebirth-AGA; p2: stillbirth-AGA vs livebirth with late FGR; p3: stillbirth-SGA vs livebirth with late FGR.

The highest incidence of disruptive (12.8% vs 0.17%;p<0.001), obstructive (58.9% vs 7.5%;p<0.001), and adaptive (56.4% vs 35.8%;p<0.001) lesions occurred in stillbirth-SGA. Moreover, in stillbirth-SGA we found a higher number of disruptive (12.8% vs 0%; p<0.001), obstructive (58.9% vs 23.5%;p<0.001) and adaptive lesions (56.4% vs 49%; p 0.47) than in livebirth-FGR.

Comparing the livebirth-FGR and livebirth-AGA groups, obstructive (23.5% vs 3.6%; p<0.001) and adaptive lesions (49% vs 34.9%; p<0.001) were still significantly higher in livebirth-FGR but lower than in stillbirth-SGA. Interestingly, comparing stillbirth-SGA and livebirth-FGR, both ≤3° customized percentile, there was a significant difference in the rate of obstructive lesions (88.2% vs 21% vs 3.6%) with livebirth-AGA.

**Fig 2:**
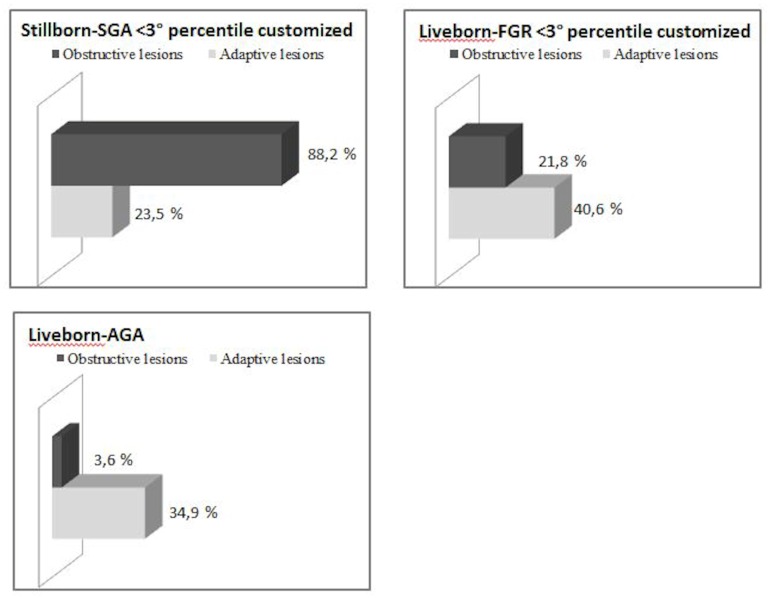
Distribution of obstructive and adaptive placental alterations in the groups.

While the ratio of obstructive and adaptive lesions for livebirth-AGA was one to ten (3.6 vs 34.9%), in unfavorable conditions, such as severe SGA or FGR, the ratio changes. In cases of severe-FGR the ratio drops to 1 to 2 (21.8% vs 40.6%) and conversely the ratio is reversed in cases of stillbirth with severe-SGA, reaching 4 to 1 (88.2% vs 23.5%) (Fig 2).

## Discussion

This is, to our knowledge, the first study that looked at a cohort of stillbirths and compared their characteristics and placental histopathology with two control groups. Fetal growth restriction, and small for gestational age as its proxy, has the strongest association with stillbirth [25]. Our study confirmed a significant relationship between SGA and risk of intrauterine death at term.

Although some risk factors for stillbirth have been identified, the main cause of stillbirth often remains elusive. Previous studies suggest that advanced maternal age is a risk factor for stillbirth [26] but in our study we do not report any significant difference between the groups. Other maternal factors like nulliparity, smoking status, pre-pregnancy BMI and obesity were all statistically significant, confirming data from other studies [25]. Concerning fetal risk factors, stillbirth is strongly related to impaired fetal growth, so both growth restriction and excess growth are risk factors for death during pregnancy [3], therefore obstetrical care should focus on both SGA and LGA pregnancies.

One challenge for our study was that it could only be conducted retrospectively: in fact, if during pregnancy we had noticed any fetuses at risk for adverse outcomes we would have changed the timing of delivery to prevent intrauterine death. In the absence of prospective information about fetal growth, the use of customized percentiles allowed us to determine retrospectively that many of these babies had failed to reach their growth potential. In previous work we have shown, using ROC curves, that customized standards identified a higher percentage of FGR in all cases of fetal death occurring after the 28th week; 26% of stillbirths occurring in the third trimester of pregnancy were reclassified as FGR by customized growth curves. [5].

An impaired placenta might become the limiting element of fetal growth. The fetus and the placenta constitute a single morpho-functional unit and must be analyzed in their ensemble. In case of poor fetal-neonatal outcomes, placental histology allows a better understanding of the pathophysiology of adverse outcomes, and provides useful information for medicolegal considerations and for the management of subsequent pregnancies.

In a review of 104 perinatal deaths, placental examination by itself could identify the cause of fetal death in 48% to 51% of cases, and placental findings could explain the cause of death in 69% of stillbirth cases [27]. These findings coincide with other reviews reporting 35% to 88% of stillbirth cases having placental findings causative of death [28]. In this study we focused on stillborn cases with a post-natal diagnosis of abnormally low fetal weight; the objective was to analyze placental characteristics, to explore if small for gestational age stillborn fetuses exhibited particular placental lesions. Comparing live-born babies from pregnancy affected by FGR and live-born from low-risk pregnancy with stillbirth, we observed an increased rate of placental lesions in stillborn cases, 14.6% vs 5.1% vs 1.9% in those with growth failure.

Obstructive lesions seem to be the main conditions associated to with low fetal weight in our stillborn cases; in fact, they were present in 54.6% of all stillbirths and 58.9% of SGA/stillbirths, while only in 7.5% of live births from low-risk pregnancy and in 23.5% of late FRG livebirths. This finding suggests that these placental abnormalities may play a crucial role. Also the relationship between obstructive lesions and adaptive lesions have a predominant function in the placentas of stillborn and liveborn with growth failure. We found adaptive lesions in more than half of SGA/stillbirths, in 49% of FGR livebirths and also in 35.8% of placentas from low-risk pregnancy. Moreover, our results show that the ratio of obstructive/adaptive lesions, and not the absolute rate of one type of these histological findings, strongly connected to adverse outcome. Chorionic villi adapt their development on the basis of environmental conditions. During chronic hypoxia placental villi could develop a heterogeneous pattern, showing accelerated maturity, small size and focal hypervascularity (chorangiosis) [29].

Unfortunately we still do not know what kind of injury plays a principal role in the placental dysfunction affecting fetal growth. In our study it is clear that obstructive lesions have a predominant role but if the placenta develops adaptive and compensatory mechanisms this can allow the fetus to survive. This hypothesis can be supported by detecting the highest number of obstructive findings in absence of an adequate number of adaptive lesions when we compare AGA stillbirths with AGA live births. Therefore the identification of this placental pattern in cases of abnormal pregnancy outcome could provide valuable information on the etiopathogenesis of the adverse event [30].

In conclusion, our study compares a population of stillbirths with live births after FGR during pregnancy, and healthy fetuses after uneventful pregnancies, analyzing the differences in placental characteristics. We confirm that several cases of stillbirth may occur in pregnancies unexpectedly affected by intrauterine growth restriction and that customized curves are the best method to assess, even retrospectively, whether a fetus has reached its growth potential. We found that placental evaluation can reveal chronic histological signs that might help to understand the pathophysiology leading to the unfavorable outcome.

## Supporting Information

S1 Table Placental characteristics in AGA and SGA stillbirth vs AGA and SGA livebirth. (DOCX)
