# Correction: Stillbirths at Term: Case Control Study of Risk Factors, Growth Status and Placental Histology

**DOI:** 10.1371/journal.pone.0213623

**Published:** 2019-03-05

**Authors:** Federico Mecacci, Caterina Serena, Laura Avagliano, Mauro Cozzolino, Eleonora Baroni, Marianna Pina Rambaldi, Serena Simeone, Francesca Castiglione, Gian Luigi Taddei, Gaetano Bulfamante

The following information is missing from the Funding statement: The authors acknowledge Fondazione Cassa di Risparmio di Pistoia e Pescia for funding the contribution of S Simeone and MP Rambaldi.

## References

[1] Mecacci F, Serena C, Avagliano L, Cozzolino M, Baroni E, Rambaldi MP, et al (2016) Stillbirths at Term: Case Control Study of Risk Factors, Growth Status and Placental Histology. PLoS ONE 11(12): e0166514 10.1371/journal.pone.0166514 27936018 PMC5147826

