# Supplementary material for: Stillbirths at Term: Case Control Study of Risk Factors, Growth Status and Placental Histology
Source: PLoS One. 2016 Dec 9;11(12):e0166514. doi: 10.1371/journal.pone.0166514 (PMC5147826; doi:10.1371/journal.pone.0166514)
Supplement: S1 Table — (DOCX) [file pone.0166514.s001.docx]

**S1 Table: Placental characteristics in AGA and SGA stillbirth vs AGA and SGA livebirth**

| **Placental lesions** | **Stillbirth-AGA (n. 95)** | **Stillbirth-SGA (n.39)** | **Livebirth-AG (n.518)** | **Livebirth-FGR (n.153)** | **p4 value** | **p5 value** | **p6 value** |
| --- | --- | --- | --- | --- | --- | --- | --- |
| **Inflammatory** | (10) 10.5% | (3) 7.7% | (24) 4.6% | (16) 10.4% | n.s. | n.s. | <0.05 |
| **Disruptive** | (5) 5.2% | (5) 12.8% | (0) 0% | (0) 0% | n.s. | <0.001 | n.s. |
| **Obstructive** | (44) 46.3% | (23) 58.9% | (19) 3.6% | (36) 23.5% | n.s. | <0.001 | <0.001 |
| **Adaptive** | (9) 9.4% | (22) 56.4% | (181) 34.9% | (75) 49% | <0.001 | <0.001 | <0.001 |

p4: stillbirth-AGA vs stillbirth-SGA; p5: stillbirth-SGA vs livebirth-AGA; p6: livebirth-AGA vs livebirth with late FGR
